# RETRACTION: Antioxidant Potential, Subacute Toxicity, and Beneficiary Effects of Methanolic Extract of Pomelo (*Citrus grandis* L. Osbeck) in Long Evan Rats

**DOI:** 10.1155/jt/9781748

**Published:** 2026-07-30

**Authors:** Journal of Toxicology

RETRACTION: M. Y. Ali, N‐E. N. Rumpa, S. Paul, et al., “Antioxidant Potential, Subacute Toxicity, and Beneficiary Effects of Methanolic Extract of Pomelo (*Citrus grandis* L. Osbeck) in Long Evan Rats,” *Journal of Toxicology* 2019, 2529569, https://doi.org/10.1155/2019/2529569.

The above article, published online on 10 June 2019 in Wiley Online Library (https://wileyonlinelibrary.com), has been retracted by agreement between the journal Editor‐in‐Chief, Dr. You‐Cheng Hseu, and John Wiley & Sons Ltd. The retraction has been agreed due to concerns identified in Figure 6, some of which have been raised on PubPeer [1]. A summary of the concerns is as follows:

• In Figure 6, there is a large region of the C‐stomach and Pomelo‐stomach panels which appears to overlap.
• In Figure 6, the C‐Brain panel appears to share an unexpected, repeated region (zoomed in) when compared to the Gp‐Brain panel in Figure 6 of [2], which shares some of the same authors.
• In Figure 6, the Pomelo‐heart panel appears identical to the Gp‐Heart panel in Figure 6 of [2].
• In Figure 6, the C‐Heart panel appears identical to the C‐Heart panel in Figure 6 of [2].
• In Figure 6, the Pomelo‐Brain panel appears to share unexpected, repeated elements with the Gp‐Brain and C‐Brain panels in Figure 6 of [2].
• In Figure 6, the Pomelo‐stomach panel appears to share unexpected, repeated elements with the C‐stomach panel in Figure 6 of [2].
• In Figure 6, the C‐pancreas panel, after 90‐degree rotation, appears highly similar to panel F in Figure 12 of [3], which shares some of the same authors.

After assessment of the concerns and the author’s response by the Chief Editor, the results and conclusions can no longer be considered reliable, and the article is therefore retracted.

Yousuf Ali agrees with the retraction; however, no response was received from the remaining authors.
